# Supplementary figures and images for: V. cholerae MakA is a cholesterol-binding pore-forming toxin that induces non-canonical autophagy
Source: J Cell Biol. 2022 Oct 4;221(12):e202206040. doi: 10.1083/jcb.202206040 (PMC9536202; doi:10.1083/jcb.202206040)

1A

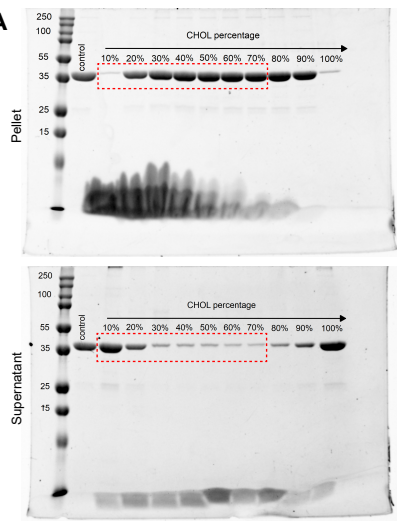

1B

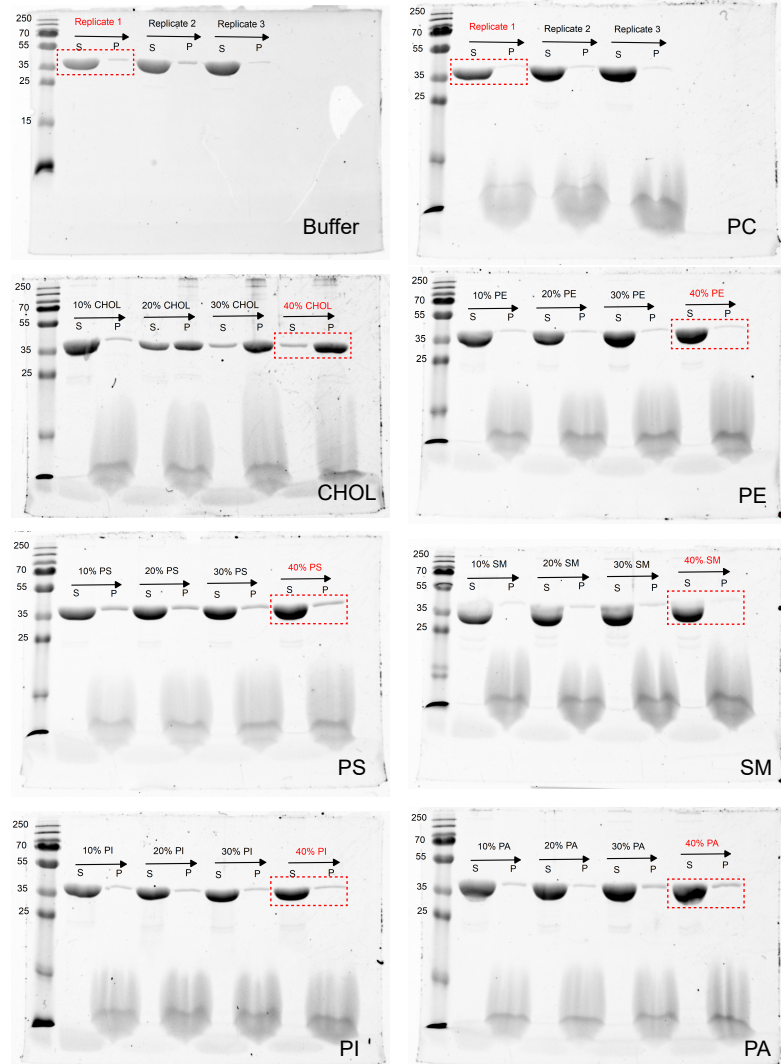

1C

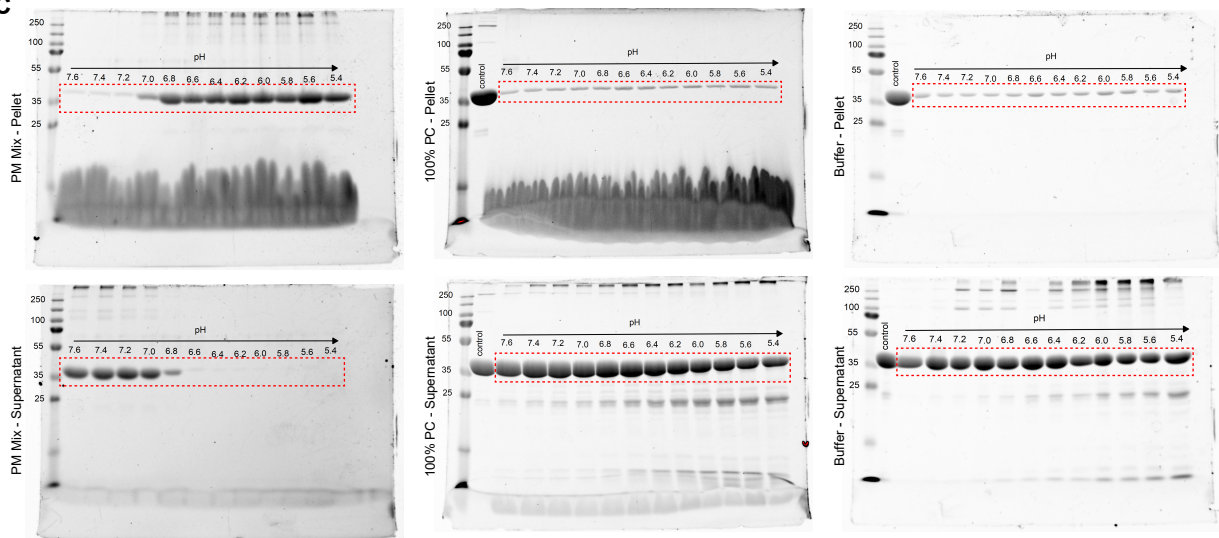

Supplement: SourceData F1 — is the source file for Fig. 1. [file JCB_202206040_SourceDataF1.pdf]

**2B**

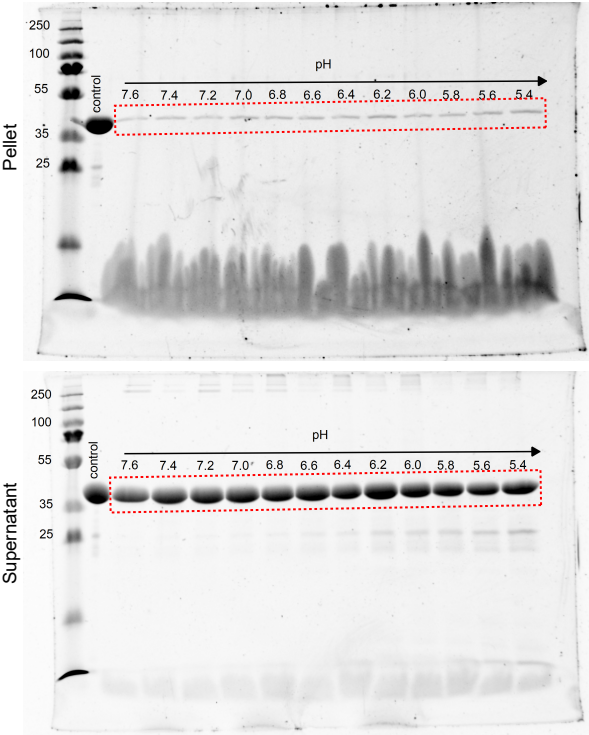

**2G**

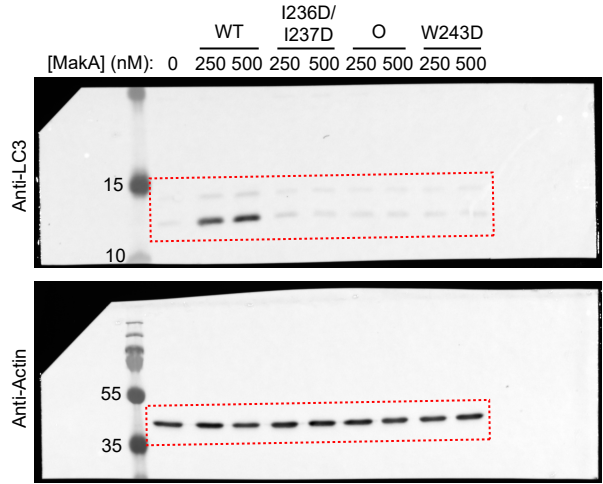

**2C**

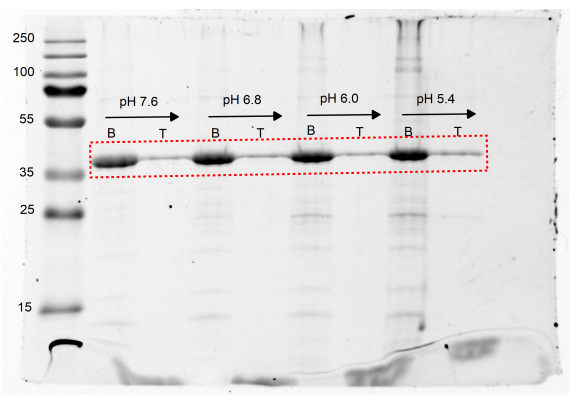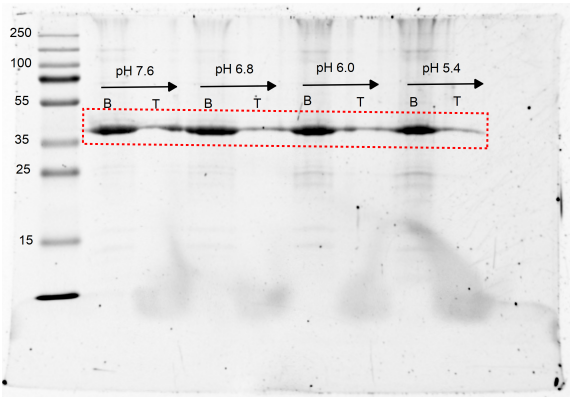

Supplement: SourceData F2 — is the source file for Fig. 2. [file JCB_202206040_SourceDataF2.pdf]

**A**

|                | WT |   |   |   | FIP200 KO |   |   |   | ATG7 KO |   |   |   |
|----------------|----|---|---|---|-----------|---|---|---|---------|---|---|---|
| MakA [125 nM]: | -  | - | + | + | -         | - | + | + | -       | - | + | + |
| Baf [250 nM]:  | -  | + | - | + | -         | + | - | + | -       | + | - | + |

| ATG9 KO |   |   |   | ATG16L1 KO |   |   |   |
|---------|---|---|---|------------|---|---|---|
| -       | - | + | + | -          | - | + | + |
| -       | + | - | + | -          | + | - | + |

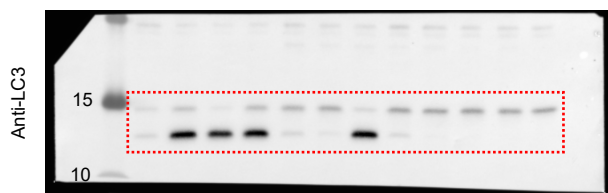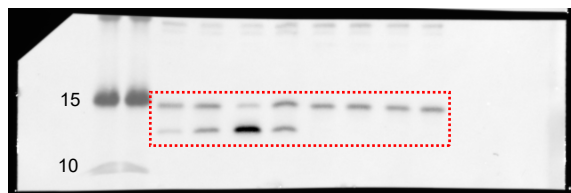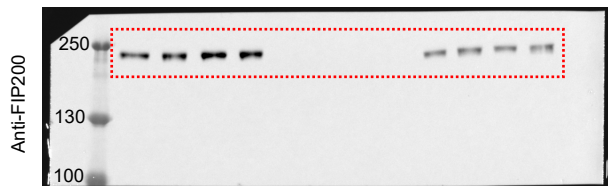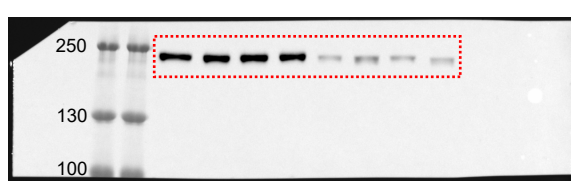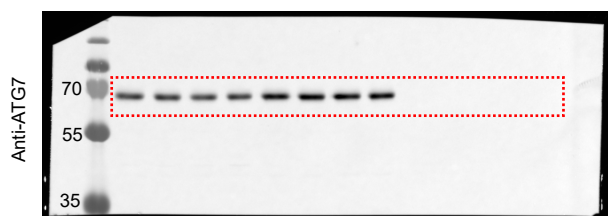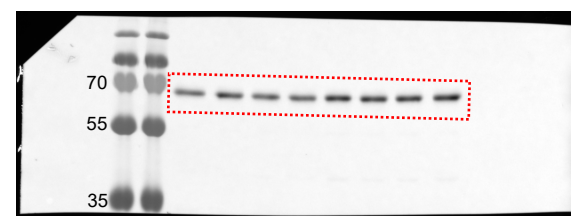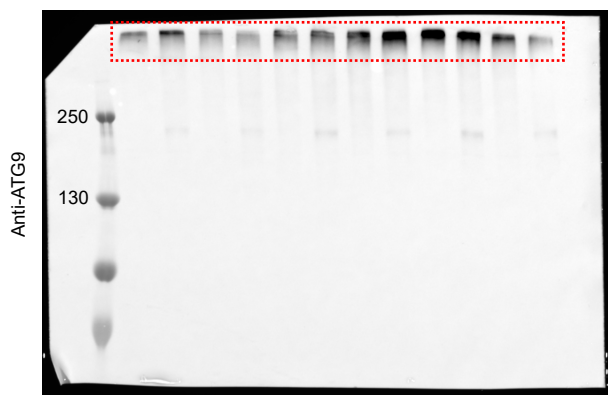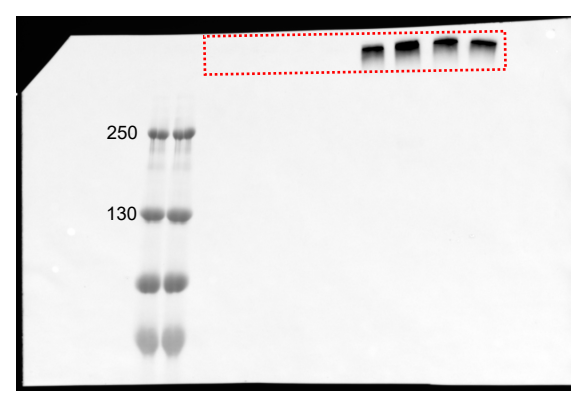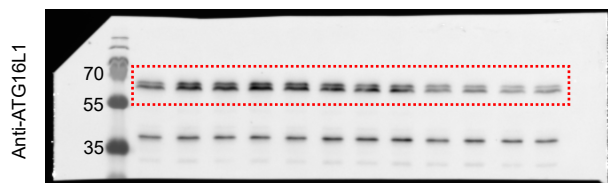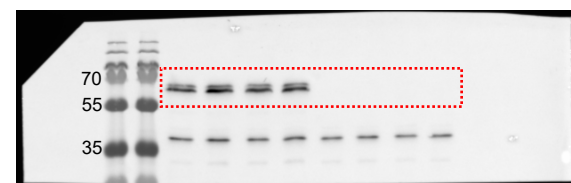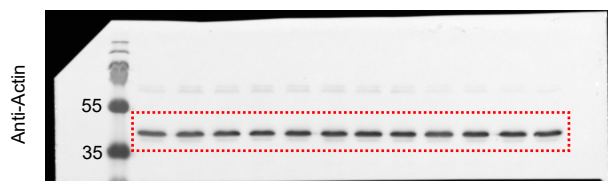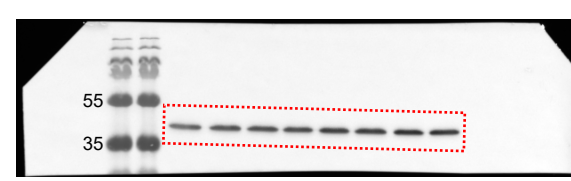

Supplement: SourceData F5 — is the source file for Fig. 5. [file JCB_202206040_SourceDataF5.pdf]

S1A

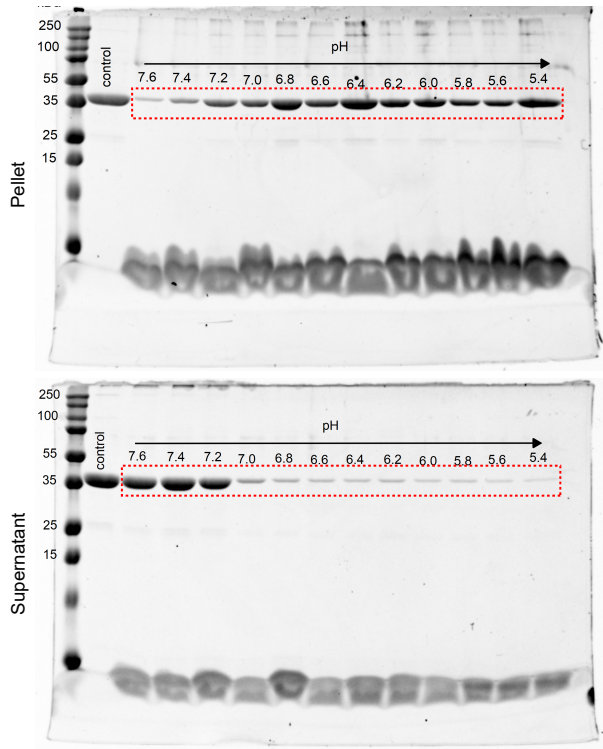

S1B

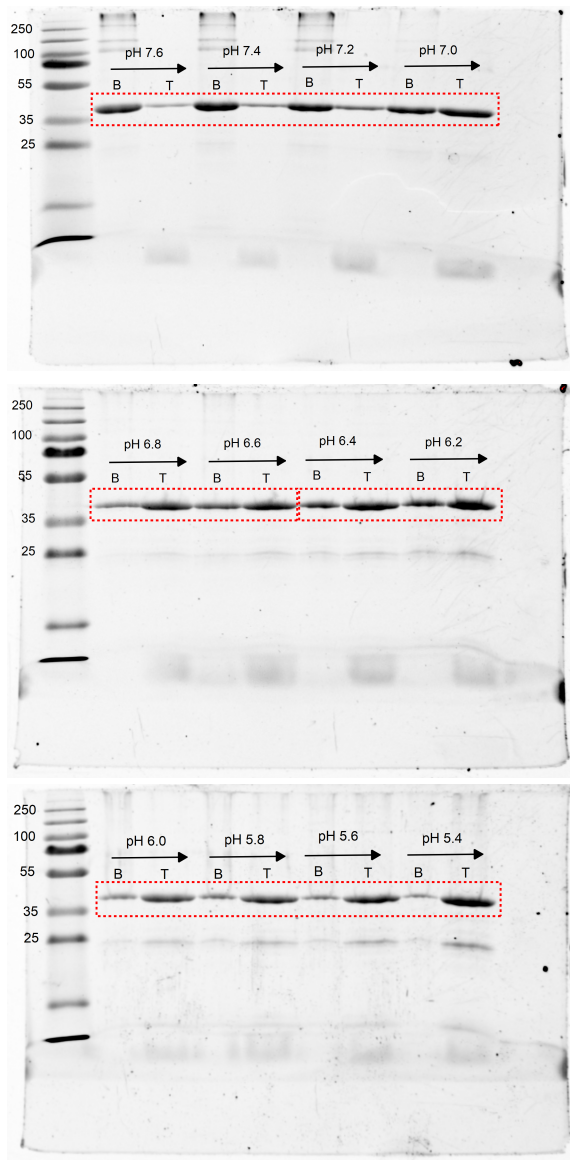

Supplement: SourceData FS1 — is the source file for Fig. S1. [file JCB_202206040_SourceDataFS1.pdf]

3B

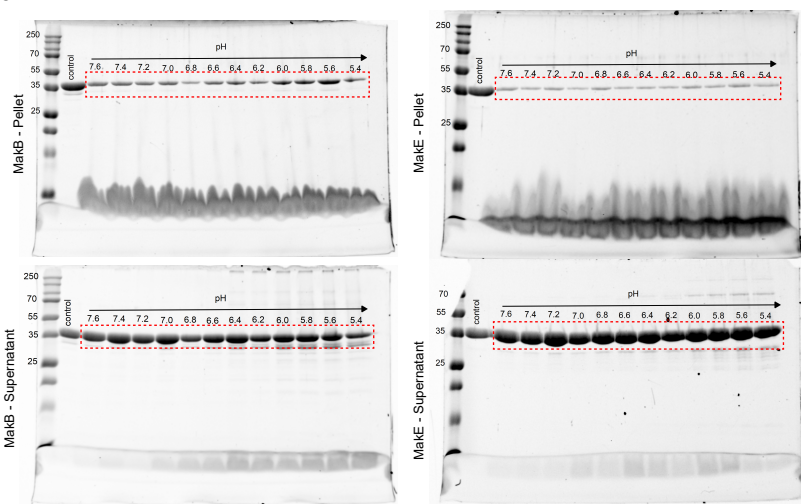

3D

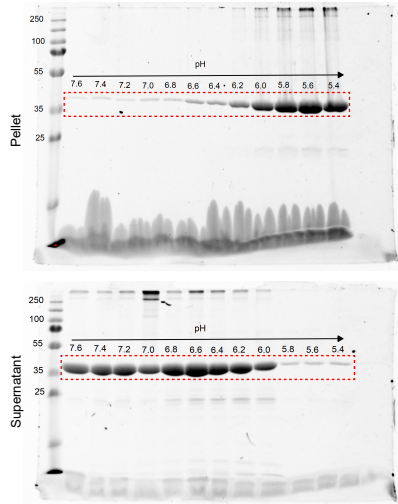

3E

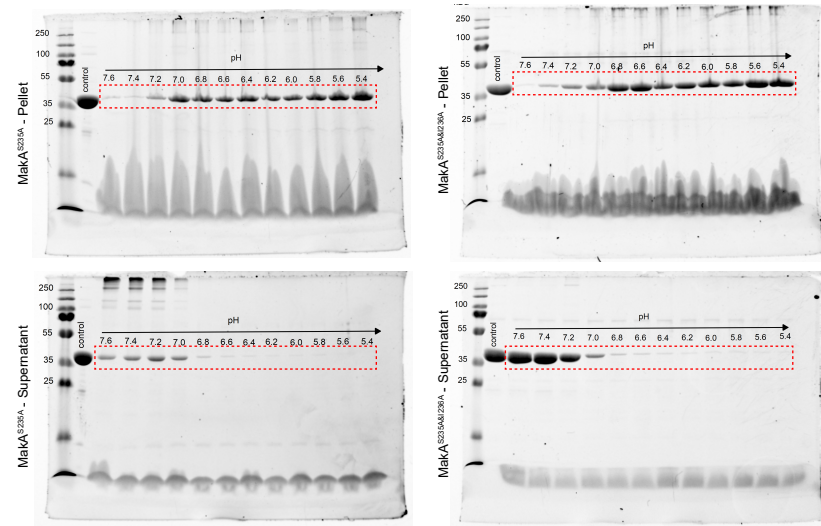

Supplement: SourceData FS3 — is the source file for Fig. S3. [file JCB_202206040_SourceDataFS3.pdf]

**4D**

SDS-PAGE

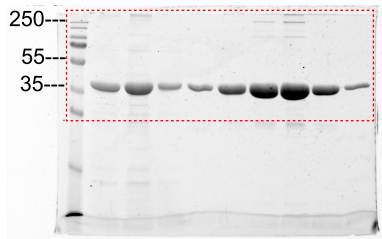

Native-PAGE

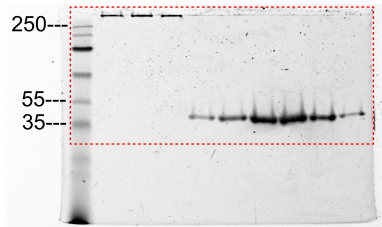**4E**

SDS-PAGE

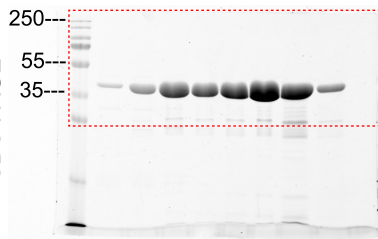

Native-PAGE

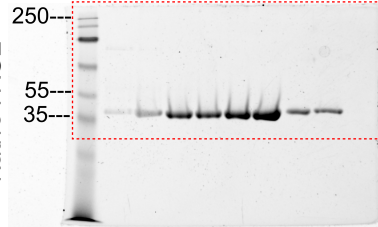**4F**

SDS-PAGE

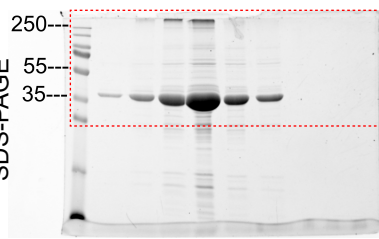

Native-PAGE

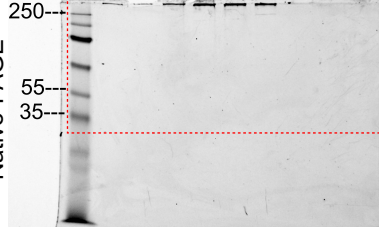

Supplement: SourceData FS4 — is the source file for Fig. S4. [file JCB_202206040_SourceDataFS4.pdf]
